# Multi-breed and multi-trait co-association analysis of meat tenderness and other meat quality traits in three French beef cattle breeds

**DOI:** 10.1186/s12711-016-0216-y

**Published:** 2016-04-23

**Authors:** Yuliaxis Ramayo-Caldas, Gilles Renand, Maria Ballester, Romain Saintilan, Dominique Rocha

**Affiliations:** GABI, INRA, AgroParisTech, Université Paris-Saclay, 78350 Jouy-en-Josas, France; Genètica i Millora Animal, IRTA, 08140 Torre Marimon, Caldes de Montbui, Spain; ALLICE, 149 rue de Bercy, 75012 Paris, France

## Abstract

**Background:**

Studies to identify markers associated with beef tenderness have focused on Warner–Bratzler shear force (WBSF) but the interplay between the genes associated with WBSF has not been explored. We used the association weight matrix (AWM), a systems biology approach, to identify a set of interacting genes that are co-associated with tenderness and other meat quality traits, and shared across the Charolaise, Limousine and Blonde d’Aquitaine beef cattle breeds.

**Results:**

Genome-wide association studies were performed using ~500K single nucleotide polymorphisms (SNPs) and 17 phenotypes measured on more than 1000 animals for each breed. First, this multi-trait approach was applied separately for each breed across 17 phenotypes and second, between- and across-breed comparisons at the AWM and functional levels were performed. Genetic heterogeneity was observed, and most of the variants that were associated with WBSF segregated within rather than across breeds. We identified 206 common candidate genes associated with WBSF across the three breeds. SNPs in these common genes explained between 28 and 30 % of the phenotypic variance for WBSF. A reduced number of common SNPs mapping to the 206 common genes were identified, suggesting that different mutations may target the same genes in a breed-specific manner. Therefore, it is likely that, depending on allele frequencies and linkage disequilibrium patterns, a SNP that is identified for one breed may not be informative for another unrelated breed. Well-known candidate genes affecting beef tenderness were identified. In addition, some of the 206 common genes are located within previously reported quantitative trait loci for WBSF in several cattle breeds. Moreover, the multi-breed co-association analysis detected new candidate genes, regulators and metabolic pathways that are likely involved in the determination of meat tenderness and other meat quality traits in beef cattle.

**Conclusions:**

Our results suggest that systems biology approaches that explore associations of correlated traits increase statistical power to identify candidate genes beyond the one-dimensional approach. Further studies on the 206 common genes, their pathways, regulators and interactions will expand our knowledge on the molecular basis of meat tenderness and could lead to the discovery of functional mutations useful for genomic selection in a multi-breed beef cattle context.

**Electronic supplementary material:**

The online version of this article (doi:10.1186/s12711-016-0216-y) contains supplementary material, which is available to authorized users.

## Background

Ruminant production is of considerable economic value, since meat and milk are important agricultural products and major sources of protein for humans. In France, beef cattle production mainly uses purebred specialized breeds such as the Charolaise, Limousine and Blonde d’Aquitaine breeds [1, 2]. As a complex phenotype, beef quality is determined by both environmental and genetic factors [3–5]. Moreover, different criteria and/or perceptions are used to define meat quality: sensory, nutritional, technological or hygienic quality. Sensory traits such as palatability, juiciness and consumer eating satisfaction depend highly on beef tenderness [6]. Several studies have focused on Warner–Bratzler shear force (WBSF) as a relevant trait to identify genetic markers associated with beef tenderness [7–9] and have led to the development of a DNA-based commercial test that targets the *calpain 1* (*CAPN1*) and *calpastatin* (*CAST*) genes. However, these markers explain only a fraction of the phenotypic variance and for some breeds (including the main French beef cattle) these tests are not informative [2, 8, 10].

Currently, there is an emerging consensus about the relevance of taking biological information into consideration for genomic selection [11, 12] and thus, it is important to identify functional single nucleotide polymorphisms (SNPs) to increase the accuracy of genomic predictions. System-based approaches have emerged as an alternative to study complex traits and to identify candidate genes. In this study, we used the association weight matrix (AWM), a systems biology approach that integrates information of genome-wide association studies (GWAS) with network inference algorithms, to identify candidate genes and their regulatory elements that could affect phenotype [13, 14]. This multi-trait approach was applied to 17 traits to identify a common set of interacting genes associated with meat tenderness and other meat quality traits across the Limousine, Charolaise and Blonde d’Aquitaine breeds.

## Methods

### Phenotypic traits, animals and genotypes

We used data resources on three French purebred specialized beef breeds, Blonde d’Aquitaine (n = 981), Charolaise (n = 1114) and Limousine (n = 1254) that were previously reported [1]. In brief, 17 traits were collected on young beef bulls: four traits were related to muscle conformation, three to carcass fatness, and 10 to meat quality (Table 1). Most of the young bulls were genotyped using the Illumina BovineSNP50 Genotyping BeadChip, i.e. 947 Blonde d’Aquitaine, 1059 Charolaise, and 1219 Limousine. All 114 sires (30, 48 and 36 for the Blonde d’Aquitaine, Charolaise, and Limousine breeds, respectively) were genotyped with the Illumina BovineHD BeadChip (777K SNPs). After quality control, 50K genotypes were imputed to HD within breed using the FImpute software [15] and pedigree information. Imputation was based on their respective reference populations of 672, 462, and 327 sires from the Charolaise, Limousine, and Blonde d’Aquitaine breeds, respectively, which were chosen based on their marginal contribution to the whole population of each breed [16].

**Table 1:** Description and summary statistics for the 17 traits analyzed in the three breeds

Trait	Acronym	Blonde d’Aquitaine			Charolaise			Limousine		
		N	Mean	SD	N	Mean	SD	N	Mean	SD
*Muscle conformation related traits*										
Live muscle score (/100)	LMS	981	58.5	10.0	1114	62.0	11.4	1254	59.5	11.5
Carcass muscle score (/18)	CMS	981	10.9	1.4	1114	10.0	1.5	1254	10.9	1.6
Carcass yield (%)	CY	981	65.0	1.5	1113	57.7	1.9	1254	62.0	1.3
Rib eye area (cm^2^)	RIBE	978	54.5	7.9	1114	53.4	8.9	1252	49.2	6.7
*Carcass fatness related traits*										
Internal cavity fat weight (kg)	CIFW	980	4.9	1.7	1107	8.7	2.2	1253	7.0	2.0
Dissected 6th rib fat (%)	RIB6	981	12.8	2.8	1113	20.4	3.9	1254	17.2	3.0
Velocity of sound^a^ (10^−3^s/cm)	VOS	964	6.253	0.017	990	6.302	0.031	1251	6.288	0.023
*Meat quality related traits*										
Warner–Bratzler shear force (N/cm^2^)	WBSF	977	40.5	11.5	1114	38.1	7.5	1252	41.0	8.4
Tenderness score (/100)	TEND	970	61.4	11.2	1113	62.4	8.4	1241	58.7	8.3
Juiciness score (/100)	JUIC	970	58.0	9.6	1113	60.0	6.6	1241	56.6	8.1
Flavor score (/100)	FLAV	970	75.8	5.5	1113	55.3	5.8	1241	59.6	6.4
Intramuscular lipid content (%)	CS	981	0.56	0.38	1114	1.53	0.86	1254	1.18	0.51
Insoluble collagen content (%)	CI	871	0.25	0.04	1114	0.30	0.05	1254	0.26	0.04
Muscle fiber section mean area (10^−6^ mm^2^)	TAMF	971	2863	594	1101	2921	820	1248	2983	686
Rib eye area/muscle fiber area (10^3^)	TFIB	968	1989	525	1101	1959	614	1246	1732	449
Muscle lightness	L^a^	979	33.1	4.4	1114	34.8	4.6	1253	32.8	4.2

^a^Average reciprocal ultra-sound speeds measured on the back, just behind the shoulder and at the 3rd lumbar [57]

### Association weight matrix approach and network analyses

After quality control, SNPs with a minor allele frequency (MAF) lower than 5 %, SNPs that mapped to the sex chromosomes or that were not mapped to the UMD3.1 bovine genome assembly were excluded. Details regarding models and the fixed effects that were fitted for each trait are available in [17]. Fifty-one GWAS were performed (17 traits for each of the three breeds) by single-trait-single-SNP association analysis, using the option *mlma* of the GCTA software [18] and the following model:

$$\tt {\mathbf{y}}_{ljm} = {\mathbf{u}}_{l} + {\mathbf{s}}_{l} {\mathbf{a}}_{k} + {\mathbf{e}}_{ljm} ,$$

where **y**_ljm_ is a vector of performance records adjusted for contemporary groups effects; **u** represents the infinitesimal genetic effect with $\tt {\mathbf{u\sim}}N(0,{\mathbf{G}}\sigma_{u}^{2} )$; **G** is the genomic relationship matrix (GRM) calculated using the autosomal SNPs based on the methodology of Yang et al. [18], with $\tt \sigma_{u}^{2}$ representing the additive genetic variance; **s**_l_ is a indicator variable depending on the l-th individual genotype for the k-th SNP, a_k_ represents the additive association of the kth SNP on the j-th trait, and **e**_ljm_ is a vector of random residual effects.

Subsequently, three independent association weight matrices (AWM) (one per breed) were built from the GWAS results. An AWM is a matrix with rows represented by genes and columns represented by phenotypes [13]. To construct an AWM, two matrices are required that both contain row-wise SNPs and column-wise phenotypes. The {*i,j*}th element of the first matrix is equal to the *p* value of the association of the i-th SNP with the j-th phenotype. In the second matrix, the {*i,j*}th element is equal to the z-score standardized additive effect of the i-th SNP for the j-th phenotype. Warner–Bratzler shear force (WBSF) was selected as the key phenotype and SNPs that were associated with WBSF (*p* value ≤0.01) were included in the AWM. In the next step, the dependency among phenotypes was explored by estimating the average number of other phenotypes (Ap) that were associated with these SNPs at a *p* value ≤ 0.01 (Ap = 2). Subsequently, all SNPs that were associated with at least two phenotypes at a *p* value ≤0.01 were included in the AWM. To build the AWM, in the next steps we followed the procedure described by Fortes et al. [13], which was modified as follows: (1) only SNPs within genes or located close to intergenic SNPs (within 10 kb of the coding region) were selected; and (2) to identify putative regulators, in addition to the transcription factors (TF) reported by Vaquerizas et al. [19], genes that encoded microRNA (miRNA) and long non-coding RNA (lnRNA) and that were mapped to the UMD 3.1 bovine genome assembly (GenBank assembly accession: GCA_000003055.3) were also considered in this analysis.

The proportion of the phenotypic variance explained by the SNPs was estimated using GCTA through a genomic restricted maximum likelihood (GREML) approach, as described in the model description. To estimate the variance explained by the AWM-SNPs, a first GRM was constructed based only on the SNPs that were selected for the AWM. Then, a second GRM was built using SNPs that were localized within genes found for all three breeds. The same numbers of randomly selected SNPs were used to made 10,000 GRM (10,000 replicates), to estimate the variance explained by those randomly selected SNPs. All these GRM were created for each breed.

Hierarchical clustering of traits (AWM columns) and genes (AWM rows) was estimated and visualized using the ‘hclust’ R function [20]. Significant gene–gene interactions (co-associations) were inferred using the partial correlation and information theory (PCIT) algorithm [21]. In the network, each node represents a gene, whereas each edge that connects two nodes represents a significant gene–gene interaction. Cytoscape software [22] was used to visualize the gene network and the CentiScaPe plug-in [23] was used to calculate specific node centrality values and network topology parameters.

### Identification of key regulators, functional classification and pathway analyses

To identify potential regulators of the candidate genes across the three breeds, we applied an information lossless approach [24] that explored the connectivity of all regulators (TF, miRNA and lnRNA) in the network. We also used the iRegulonv1.3 Cytoscape plugin [25] to in silico identify TF binding site motifs in the *cis*-regulatory elements that were shared among the identified common candidate genes. Gene functional classification and pathway analyses were performed using Ingenuity Pathways Analysis software (IPA; Ingenuity Systems, Redwood City, CA). Over-represented gene ontology (GO) terms were identified using ClueGO, Cytoscape plug-in [26]. The cut-off for considering a significant over-representation was established by Benjamini and Hochberg multiple-test correction [27] (*p* value ≤0.05).

## Results and discussion

### Phenotype statistics and GWAS results

We used a systems biology approach to identify candidate genes that were co-associated with WBSF and 16 other phenotypes across the Charolaise, Limousine and Blonde d’Aquitaine breeds. Summary statistics, number of records available for each trait, and a brief description of the 17 traits are in Table 1. GWAS were performed for these 17 traits using the BovineHD BeadChip (777K) genotypes for each of the three breeds. The results of these GWAS served as the basis for the AWM approach and other analyses which are discussed below.

### Single-breed analyses

After quality control, 533,604 (Blonde d’Aquitaine), 539,337 (Limousine) and 543,682 (Charolaise breed) informative SNPs remained for analysis. After applying a further set of selection criteria (see Methods section), the numbers of SNPs that were retained to build the AWM were equal to 2339 (Blonde d’Aquitaine), 2331 (Limousine) and 2518 (Charolaise). Cluster distributions were in agreement with the physiological similarities and genetic correlation among traits (see Additional file 1: Figure S1). Hence, a clear separation between WBSF and sensory traits such as tenderness sensory score (TEND), juiciness sensory score (JUIC) and flavor sensory score (FLAV) was observed. Opposite directionality of the estimated additive values was also observed between WBSF, intramuscular composition (IMF), fatness and conformations traits (see Additional file 1: Figure S1). In agreement with previous studies [28], SNPs detected with the AWM approach explained between 68 and 74 % of the phenotypic variance for WBSF (Table 2). Moreover, previously estimated genetic correlations agreed moderately well with AWM-based correlations (see Additional file 2: Table S1).

**Table 2:** Proportion of phenotypic variance explained by SNPs identified with the AWM approach, by the 206 common genes, and by the 206 randomly selected SNPs

Breed	AWM SNPs	206 common genes	206 random SNPs
Limousine	0.74 ± 0.03	0.28 ± 0.03	0.04 ± 0.02
Charolaise	0.68 ± 0.04	0.28 ± 0.03	0.05 ± 0.02
Blonde d’Aquitaine	0.72 ± 0.04	0.30 ± 0.04	0.09 ± 0.03

### Breed-specific candidate genes

Consistent with previous reports [13, 29], which support the reliability of our results, relevant biological information was captured by the within-breed AWM. SNPs that map to well-known candidate genes for tenderness and meat quality traits were identified (Table 3). Most of these SNPs were breed-specific in agreement with [17]. For example, using the AWM approach, we identified genes that encode proteins displaying differential abundance in the muscle of animals with extreme meat tenderness phenotypes [30]. These included for the Charolaise breed: *phosphoglucomutase 1* (*PGM1*), *stress*-*induced phosphoprotein 1* (*STIP1*) and *capping protein muscle Z*-*line, beta* (*CAPZB*); for the Blonde d’Aquitaine breed: *ankyrin 1 erythrocytic* (*ANK1*), *Ki*-*67* (*MKI67*) and *myosin regulatory light chain 2 skeletal muscle isoform* (*MYLPF*); and for the Limousine breed: *glutathione S*-*transferase alpha 4 and 5* (*GSTA4*, *GSTA5*), *leptin* (*LEP*) and *fatty acid binding protein 4* (*FABP4*).

**Table 3:** Candidate genes identified for meat tenderness and related meat quality traits by breed and across breeds

Breed	Candidate gene
Limousine	*Glutathione S*-*transferase alpha 4* (*GSTA4*)
	*Glutathione S*-*transferase alpha 5* (*GSTA5*)
	*Leptin* (*LEP*)
	*Fatty acid binding protein 4* (*FABP4*)
Charolaise	*Homogentisate 1,2*-*dioxygenase* (*HGD*)
	*Lysyl oxidase* (*LOX*)
	*Somatostatin* (*SST*)
	*Phosphoglucomutase 1* (*PGM1*)
	*Stress*-*induced phosphoprotein 1* (*STIP1*)
	*Capping protein muscle Z*-*line, beta* (*CAPZB*)
Blonde d’Aquitaine	*Ankyrin 1* (*ANK1*)
	*Ki*-*67* (*MKI67*)
	*Myosin regulatory light chain 2, skeletal muscle isoform* (*MYLPF*)
	*Transcription termination factor, RNA polymerase I* (*TTF1*)
Between two breeds	*Myostatin* (*MSTN*)
	*Calpastatin* (*CAST*)
	*Calpain 5* (*CAPN5*)
	*Growth hormone receptor* (*GHR*)
	*RAR*-*related orphan receptor C* (*RORC*)
	*ArfGAP with SH3 domain, ankyrin repeat and PH domain 1* (*ASAP1*)
	*Thyroglobulin* (*TG*)
	*Calpain 1* (*CAPN1*)
Across-breeds	*Collagen type XI alpha 1* (*COL11A1*)
	*RAB11 family interacting protein 5* (*RAB11FIP5*)

### Breed-specific networks and regulators

Gene–gene interactions were predicted using PCIT [21] and three marker-derived gene networks (one per breed) were inferred. Predicted interactions were based on partial correlations between SNP effects across traits that were detected as significant by the PCIT algorithm. To facilitate a posteriori analysis, network reduction was applied by considering only correlations that were greater than the mean ± 2 × SD in absolute value (r_Blonded’Aquitaine_ ≥ 0.83, r_Charolaise_ ≥ 0.84, r_Limousine_ ≥ 0.83). Topological parameters were similar across the three breeds and the numbers of nodes connected by the largest correlations were equal to 2063 nodes connected by 8869 edges for the Blonde d’Aquitaine breed, 2116 nodes connected by 10,248 edges for the Charolaise breed, and 1979 nodes connected by 5734 edges for the Limousine breed.

To identify potential regulators, we focused on TF, miRNA and lnRNA. In agreement with its relevant role as a regulator of muscle cell growth and differentiation, *myostatin* (*MSTN*) was identified among the top TF for two of the three breeds (Charolaise and Blonde d’Aquitaine). Specifically for the Blonde d’Aquitaine breed, *MSTN* co-associated with the largest number of traits (eight traits, including WBSF), while *MSTN* co-associated with five traits for the Charolaise breed. Moreover, for both these breeds, a co-operative role of *MSTN* with two different miRNA was predicted in the transcriptional regulation of tenderness and meat quality traits: *bta*-*mir*-*488* for Charolaise and *ENSBTAG00000037365* for Blonde d’Aquitaine. Among the top regulators identified for the Limousine breed, we found the *proline, glutamate and leucine rich protein 1* (*PELP1*) and the *cAMP responsive element binding protein 3*-*like 3* (*CREB3L3*) genes. CREB3L3 is a TF that is activated by cyclic AMP stimulation and is linked to triglyceride metabolism and acute inflammatory response [31]. Interestingly, a relationship between acute stress and WBSF has been reported in Angus cattle [31]. PELP1 is a member of the chromatin remodeling complexes and a coactivator of the estrogen receptor and it plays an important role in the ER/growth factor cross-talk [32].

### Multi-breed analyses

**Fig. 1:**
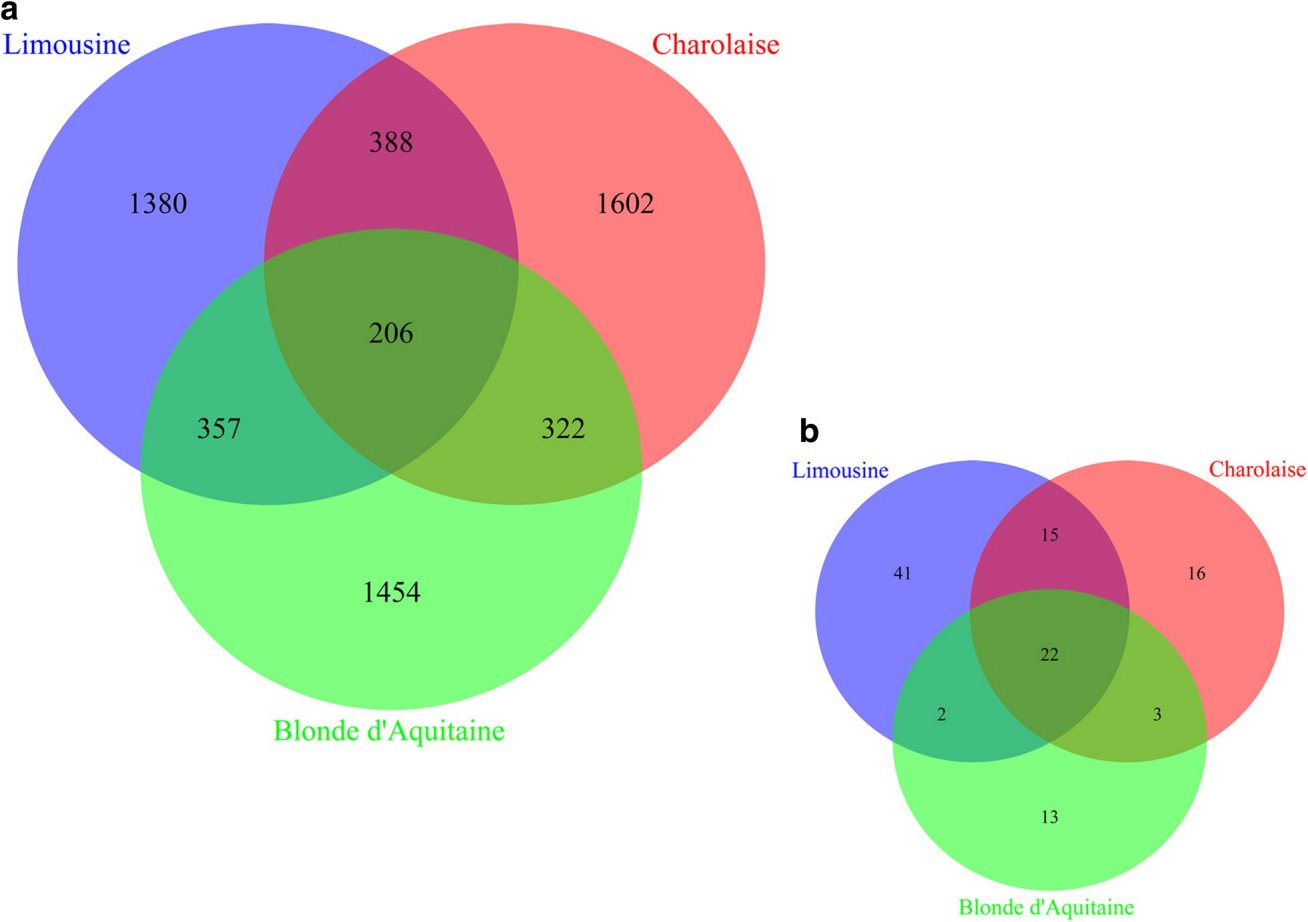
Number of overlapping genes (**a**) and overlapping GO biological processes (**b**)

Beyond identifying within-breed candidate genes, the main goal of our study was to identify a common set of interacting genes, biological pathways and functions associated with meat tenderness and meat quality across the three breeds. Therefore, comparisons at the AWM and functional levels were performed as follows. At the gene level, the AWM approach revealed only a few common genes across the three breeds, their number decreasing from ~22 % of the total number of genes between two breeds to only ~8 % across the three breeds (representing 206 genes) (Fig. 1a). Previous studies had already reported that the number of quantitative trait locus (QTL) regions that overlap between bovine breeds is small [33, 34]. It should be noted that the number of common SNPs identified by the AWM approach was very small, ranging from 37 to 48 SNPs between two breeds, and not a single common SNP was identified when all three breeds were compared (see Additional file 3: Figure S2A). This can be explained by breed differences in allele frequencies and/or linkage disequilibrium (LD) and also by the possibility of different mutations in the same candidate gene between breeds. In agreement with previous reports, there was more overlap at the pathway and GO functional levels [35]. We found 24 over-represented pathways and 22 GO terms that were shared between the three breeds (see Additional file 3: Figure S2A) and Fig. 1b, including biological processes such as cell morphogenesis involved in differentiation (GO:0000904), calcium ion transmembrane transport (GO:0070588), Rho protein signal transduction (GO:0007266), positive regulation of GTPase activity (GO:0043547) and Rac protein signal transduction (GO:0016601) (see Additional file 4: Table S2).

### Across-breed candidate genes

**Fig. 2:**
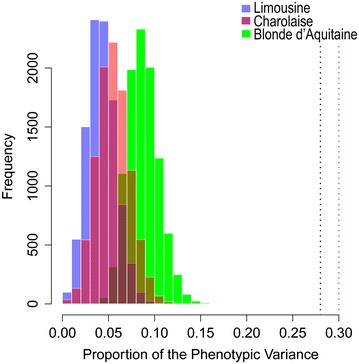
Distribution of the proportion of phenotype variance for WBSF explained by 206 randomly selected SNPs in 10,000 replicates. The *black vertical line* represents the proportion of the phenotype variance for WBSF explained by the 206 common SNPs identified for the Limousine and Charolaise breeds. The *green vertical line* represents the proportion of the phenotype variance for WBSF explained by the 206 common SNPs identified for the Blonde d’Aquitaine breed

Among the candidate genes that have been reported to be associated with meat quality traits, *calpastatin* (*CAST*), *calpain5* (*CAPN5*), *growth hormone receptor* (*GHR*), *RAR*-*related orphan receptor C* (*RORC*), *myostatin* (*MSTN*), and *ArfGAP with SH3 domain, ankyrin repeat and PH domain 1* (*ASAP1*) were identified in at least two of the three breeds (Table 3), and *calpain 1* (*CAPN1*), *collagen type XI alpha 1* (*COL11A1*), and *RAB11 family interacting protein 5* (*RAB11FIP5*) were identified in all three breeds. The estimated proportion of phenotypic variance for WBSF explained by the SNPs that mapped to the 206 genes that were common across the three breeds ranged from 28 to 30 % and was significantly larger (*p* value <0.01) than the variance explained by the same number of randomly (10,000 replicates) selected SNPs (Table 2; Fig. 2).

To identify other candidate genes, we examined the position of each SNP located within the 206 genes against the bovine QTL database (http://www.animalgenome.org/cgi-bin/QTLdb/BT/index). In spite of differences in the genetic background of the animals included in our study and in the methods used to measure WBSF, there were several overlapping QTL for WBSF between our study and those previously reported across five taurine cattle breeds [8] (see Additional file 5: Table S3). We identified several interesting candidate genes within these QTL regions, i.e. the *RNA binding motif protein 20* (*RBM20*) gene, which is predominantly expressed in striated muscle and the expression of which correlates with sarcomere assembly [36]. Genes involved in adipogenesis (*EBF1*), calcium metabolism (*MRVI1*, *KCNIP4*, *PCDH7*, and *CUBN*), muscle metabolism, growth retardation and bone metabolism (*WWOX* and *AKAP6*) were also observed.

It is interesting to note that 29 % (60/206) of the common genes that we detected in our multi-breed analysis have been reported to be associated with WBSF in Brazilian Nelore beef cattle using a Bayesian approach [37] (see Additional file 6: Table S4) and ~39 % (80/206) of the common genes have been reported to be associated with intramuscular fat (IMF) deposition in Australian beef cattle [38] (see Additional file 7: Table S5). Oury et al. [39] reported the existence of a relationship of meat tenderness with IMF content, as well as with total and insoluble collagen content, glycolytic metabolism and mean cross-sectional fibre area. IMF explains part of the variability in sensory tenderness score of *longissimus dorsi* muscle in beef cattle [4]. In total, 23 genes were common across the Brazilian, Australian and French datasets (see Additional file 8: Table S6). This overlap of genes and QTL for WBSF suggests that some of the identified candidate genes may be useful in multi-breed gene-assisted selection programs for meat tenderness. Further studies to identify the functional mutations are warranted.

### Key regulators across breeds

Among the 206 genes, 22 were predicted as putative regulators (21 TF + 1 miRNA). Analysis of the literature showed that some of these 22 putative regulators belong to biological pathways and relevant functions that are related to muscle development. For example, *RAR*-*related orphan receptor A* (*RORA*) encodes a nuclear receptor that is essential for the activation of myogenic-specific markers and regulates a number of genes involved in lipid metabolism [40, 41]. *Zinc finger protein 423* (*ZNF423*) encodes a TF that is involved in metal ion binding and was recently reported as a potential candidate gene for skeletal muscle growth rate and major cell types in cattle [42]. Regulators involved in epigenetic modifications, such as *histone deacetylase 4* (*HDAC4*), were also identified. *HDAC4* is associated with cell differentiation and tissue development and interestingly, it was shown to be involved in muscle maturation via an interaction with the myocyte enhancer factor [43, 44]. We identified only one microRNA among the common regulators, i.e. *bta*-*let*-*7i*, which was initially characterized from bovine adipose tissue and mammary gland [45]. This microRNA maps to bovine chromosome 5 (between 51,209,080 and 51,209,164 bp) and, to the best of our knowledge, there is no report on an association of *bta*-*let*-*7i* with meat quality traits.

To detect potential candidate regulators among the 206 common genes that were identified in our study, we performed in silico identification of enriched TF binding motifs and TF with the iRegulon Cytoscape plugin [25]. We detected different enriched motifs that can bind directly to candidate TF (see Additional file 9: Table S7) such as the *interferon regulatory factor 1* (*IRF1*) TF (enriched motif ID = transfac_pro-M00747; NES = 3.860; Targets = 57), which was previously identified as a master regulator in the bovine skeletal muscle of two beef cattle breeds (Piedmontese x Hereford and Wagyu x Hereford) [46]; the *nuclear factor of activated T*-*cells, cytoplasmic, calcineurin*-*dependent 4* (*NFATC4*) TF (enriched motif ID = transfac_pro-M01734; NES = 3.717, Targets = 50), which encodes a member of the nuclear factor of activated T cells (NFAT) DNA-binding transcription complex protein family that plays an important role in skeletal muscle development and growth [47] and in glucose and insulin homeostasis [48]; the *core*-*binding factor, beta subunit* (*CBFB*) TF (enriched motif ID = hdpi-CBFB; NES = 3.339; Targets = 43), which encodes a beta subunit of a heterodimeric *PEBP2/CBF* TF family that regulates genes involved in osteogenesis [49]; the *serum response factor* (*SRF*) (enriched motif ID = yetfasco-145; NES = 3.295; Targets = 23), the *adenosine deaminase, RNA*-*specific, B1* (*ADARB1*) (enriched motif ID = hdpi-ADARB1; NES = 3.231; Targets = 63) and the *mex*-*3 RNA binding family member C* (*MEX3C*) (enriched motif ID = hdpi-RKHD2; NES = 3.339; Targets = 43), which play a crucial role in skeletal muscle growth and maturation [50], skeletal myogenesis [51], and postnatal growth and energy balance regulation [52, 53], respectively. Finally, iRegulon identified the *GATA binding protein 2* (*GATA2*) (NES = 4.887) as an enriched TF that can bind 73 of the 206 analyzed genes. Interestingly, GATA factors play a key role in the regulation of adipogenesis and GATA2 is included in the top TF that were detected in a genome-wide analysis aimed at linking transcriptional and regulatory information in bovine skeletal muscle [54].

### Implications and conclusions

Consumer eating satisfaction and palatability attributes depend highly on meat tenderness and, thus, improvement of meat tenderness is relevant to the beef industry. In this study, we used a systems biology approach to explore dependencies among traits, identify pleiotropic SNPs, and infer networks of interacting genes that affect correlated traits. Genetic heterogeneity among breeds was observed and most SNPs were significantly associated with WBSF within rather than across breeds, with only 8 % of the total number of genes revealed by the AWM approach found to be co-associated in all three breeds. Compared to the within-breed AWM, the common set of genes explained between 38 and 42 % of the phenotypic variance (Table 2). This decrease in % of variance explained is expected as a consequence of the exclusion of relevant breed-specific variants.

Multivariate approaches that exploit correlations between traits have been used, for example in humans, to increase the prediction accuracy for schizophrenia, bipolar disorder, and major depressive disorder [55]. Using the AWM approach, Snelling et al. [28] reported that the accuracy of functionally-informed genomic predictions is higher than that of classical genomic predictions for beef tenderness. In addition, in agreement with recent reports [11], Snelling et al. [28] underline the importance of properly choosing the a priori biological information to obtain an increase in prediction accuracy. Our results also suggest that breed-specific mutations may affect differently the same genes. Therefore, it is likely that, depending on allele frequencies and LD patterns, a marker that is identified to be associated with a trait for one breed may not be informative for another breed. We hypothesize that for multi-breed programs, strategies that use the LD between markers (such as the use of haplotypes), together with functional information, will be more accurate for genomic predictions than using functionally-guided single-markers.

We demonstrate that the use of systems biology approaches that explore the association between correlated traits can increase the power to identify candidate genes beyond the classical one-dimensional approach also in a multi-breed context. However, it is possible that other important SNPs for meat tenderness were not captured by our approach, for example, because their impact is breed-specific. In this study, we implemented the AWM approach in a *cis* gene-centered manner. As recently demonstrated by the human ENCODE project, most biologically meaningful variants are likely to have a regulatory function, which suggests that most causative SNPs will be non-coding [56]. The same is expected for other species, including cattle. Improvement of the functional annotation of the bovine genome should facilitate the identification of functional mutations. Studies to better understand the genetic basis of complex traits is an active area of research and a priority to improve the accuracy of genomic predictions. In this regard, the use of integrative approaches that combine multiple sources of complementary information (e.g. intermediate phenotypes) could improve our understanding of the biological processes underlying phenotypes of interest to support technological development towards the improvement of beef quality.

## Additional files

10.1186/s12711-016-0216-y Hierarchical cluster analysis for the Limousine, Charolaise and Blonde d'Aquitaine breeds. The figure represents the distribution of clusters for the 17 traits analyzed on the x-axis and the distribution of clusters for co-associated genes on the y-axis. Abbreviations for traits are described in Table 1.
10.1186/s12711-016-0216-y Comparison of genetic correlations between traits and SNP-based correlations (777K, AWM and 206 common genes). The data summarize the comparison between genetic correlations and SNP-based correlations across the three beef cattle breeds.
10.1186/s12711-016-0216-y (A) Number of overlapping SNPs. (B) Overlapping pathways. The figure represents the number of overlapping SNPs and pathways identified across the three beef cattle breeds.
10.1186/s12711-016-0216-y Description of the 22 common overrepresented GO biological processes identified across the three breeds.
10.1186/s12711-016-0216-y Description of the genes that overlap with the QTL for Warner-bratzler shear force (WBSF) according to the Cattle QTLdb.
10.1186/s12711-016-0216-y List of 60 common genes that are associated with WBSF in Brazilian Nelore beef cattle. The data describe the 60 common genes that were previously reported to be associated with WBSF in Brazilian Nelore beef cattle.
10.1186/s12711-016-0216-y List of 80 common genes that are associated with intramuscular fat deposition in Australian beef cattle.
10.1186/s12711-016-0216-y Description of the 23 common genes across the Brazilian, Australian and French datasets.
10.1186/s12711-016-0216-y Enriched TF binding motifs and candidate TF that can bind these motifs identified by iRegulon (default parameters) for the 206 common genes. The data describe the in silico identified TF binding sites motifs in the cis-regulatory elements of the co-associated genes identified across the three breeds. The TF that present a direct motif similarity and are functionally related with skeletal muscle development are highlighted in black.

## References

[1] Allais S, Levéziel H, Payet-Duprat N, Hocquette JF, Lepetit J, Rousset S (2010). The two mutations, Q204X and nt821, of the *myostatin* gene affect carcass and meat quality in young heterozygous bulls of French beef breeds. J Anim Sci.

[2] Allais S, Journaux L, Levéziel H, Payet-Duprat N, Raynaud P, Hocquette JF (2011). Effects of polymorphisms in the *calpastatin* and *µ*-*calpain* genes on meat tenderness in 3 French beef breeds. J Anim Sci.

[3] Warner RD, Greenwood PL, Pethick DW, Ferguson DM (2010). Genetic and environmental effects on meat quality. Meat Sci.

[4] Chriki S, Renand G, Picard B, Micol D, Journaux L, Hocquette JF (2013). Meta-analysis of the relationships between beef tenderness and muscle characteristics. Livest Sci.

[5] Weston AR, Rogers RW, Althen TG (2002). Review: the role of collagen in meat tenderness. Prof Anim Sci.

[6] Huffman KL, Miller MF, Hoover LC, Wu CK, Brittin HC, Ramsey CB (1996). Effect of beef tenderness on consumer satisfaction with steaks consumed in the home and restaurant. J Anim Sci.

[7] Barendse W, Harrison BE, Bunch RJ, Thomas MB (2008). Variation at the *Calpain 3* gene is associated with meat tenderness in zebu and composite breeds of cattle. BMC Genet.

[8] McClure MC, Ramey HR, Rolf MM, McKay SD, Decker JE, Chapple RH (2012). Genome-wide association analysis for quantitative trait loci influencing Warner–Bratzler shear force in five taurine cattle breeds. Anim Genet.

[9] Hulsman Hanna LL, Garrick DJ, Gill CA, Herring AD, Riggs PK, Miller RK (2014). Genome-wide association study of temperament and tenderness using different Bayesian approaches in a Nellore Angus crossbred population. Livest Sci.

[10] Van Eenennaam AL, Li J, Thallman RM, Quaas RL, Dikeman ME, Gill CA (2007). Validation of commercial DNA tests for quantitative beef quality traits. J Anim Sci.

[11] Perez-Enciso M, Rincon J, Legarra A (2015). Sequence- vs. chip-assisted genomic selection: accurate biological information is advised. Genet Sel Evol.

[12] Meuwissen T, Goddard M (2010). Accurate prediction of genetic values for complex traits by whole-genome resequencing. Genetics.

[13] Fortes MRS, Reverter A, Zhang Y, Collis E, Nagaraj SH, Jonsson NN (2010). Association weight matrix for the genetic dissection of puberty in beef cattle. Proc Nat Acad Sci USA.

[14] Reverter A, Fortes MS (2013). Association weight matrix: a network-based approach towards functional genome-wide association studies. Methods Mol Biol.

[15] Sargolzaei M, Chesnais JP, Schenkel F (2014). A new approach for efficient genotype imputation using information from relatives. BMC Genom.

[16] Hoze C, Fouilloux MN, Venot E, Guillaume F, Dassonneville R, Fritz S (2013). High-density marker imputation accuracy in sixteen French cattle breeds. Genet Sel Evol.

[17] Allais S, Levéziel H, Hocquette JF, Rousset S, Denoyelle C, Journaux L (2014). Fine mapping of quantitative trait loci underlying sensory meat quality traits in three French beef cattle breeds. J Anim Sci.

[18] Yang J, Lee SH, Goddard ME, Visscher PM (2011). GCTA: a tool for genome-wide complex trait analysis. Am J Hum Genet.

[19] Vaquerizas JM, Kummerfeld SK, Teichmann SA, Luscombe NM (2009). A census of human transcription factors: function, expression and evolution. Nat Rev Genet.

[20] Ihaka R, Gentleman R (1996). R: a language for data analysis and graphics. J Comput Graph Stat.

[21] Reverter A, Chan EKF (2008). Combining partial correlation and an information theory approach to the reversed engineering of gene co-expression networks. Bioinformatics.

[22] Shannon P, Markiel A, Ozier O, Baliga NS, Wang JT, Ramage D (2003). Cytoscape: a software environment for integrated models of biomolecular interaction networks. Genome Res.

[23] Scardoni G, Petterlini M, Laudanna C (2009). Analyzing biological network parameters with CentiScaPe. Bioinformatics.

[24] Reverter A, Fortes MRS (2013). Breeding and genetics symposium: building single nucleotide polymorphism-derived gene regulatory networks: towards functional genomewide association studies. J Anim Sci.

[25] Janky R, Verfaillie A, Imrichova H, Van de Sande B, Standaert L, Christiaens V (2014). iRegulon: from a gene list to a gene regulatory network using large motif and track collections. PLoS Comput Biol.

[26] Bindea G, Mlecnik B, Hackl H, Charoentong P, Tosolini M, Kirilovsky A (2009). ClueGO: a cytoscape plug-into decipher functionally grouped gene ontology and pathway annotation networks. Bioinformatics.

[27] Benjamini Y, Hochberg Y (1995). Controlling the false discovery rate: a practical and powerful approach to multiple testing. J R Stat Soc Ser B.

[28] Snelling WM, Cushman RA, Keele JW, Maltecca C, Thomas MG, Fortes MRS (2013). Breeding and genetics symposium: networks and pathways to guide genomic selection. J Anim Sci.

[29] Widmann P, Reverter A, Fortes MR, Weikard R, Suhre K, Hammon H (2013). A systems biology approach using metabolomic data reveals genes and pathways interacting to modulate divergent growth in cattle. BMC Genomics.

[30] Chaze T, HocquetteJF Meunier B, Renand G, Jurie C, Chambon C, Toldra F, Nollet LML (2013). Biological markers for meat tenderness of the three main French beef breeds using 2-DE and MS approach. Proteomic in foods: principles and applications.

[31] Zhao C, Tian F, Yu Y, Luo J, Mitra A, Zhan F (2012). Functional genomic analysis of variation on beef tenderness induced by acute stress in Angus cattle. Comp Funct Genomics.

[32] Nagpal JK, Nair S, Chakravarty D, Rajhans R, Pothana S, Brann DW (2008). Growth factor regulation of estrogen receptor coregulator PELP1 functions via protein kinase A pathway. Mol Cancer Res.

[33] Saatchi M, Schnabel RD, Taylor JF, Garrick DJ (2014). Large-effect pleiotropic or closely linked QTL segregate within and across ten US cattle breeds. BMC Genomics.

[34] Raven LA, Cocks BG, Hayes BJ (2014). Multibreed genome wide association can improve precision of mapping causative variants underlying milk production in dairy cattle. BMC Genomics.

[35] Flori L, Fritz S, Jaffrezic F, Boussaha M, Gut I, Heath S (2009). The genome response to artificial selection: a case study in dairy cattle. PLoS One.

[36] Guo W, Schafer S, Greaser ML, Radke MH, Liss M, Govindarajan T (2012). *RBM20*, a gene for hereditary cardiomyopathy, regulates titin splicing. Nat Med.

[37] Tizioto PC, Decker JE, Taylor JF, Schnabel RD, Mudadu MA, Silva FL (2013). Genome scan for meat quality traits in Nelore beef cattle. Physiol Genomics.

[38] Ramayo-Caldas Y, Fortes MRS, Hudson NJ, Porto-Neto LR, Bolormaa S, Barendse W (2014). A marker-derived gene network reveals the regulatory role of *PPARGC1A*, *HNF4G*, and *FOXP3* and in intramuscular fat deposition of beef cattle. J Anim Sci.

[39] Oury MP, Picard B, Briand M, Blanquet JP, Dumont R (2009). Interrelationships between meat quality traits, texture measurements and physicochemical characteristics of M. rectus abdominis from Charolais heifers. Meat Sci.

[40] Raspe E, Duez H, Gervois P, Fievet C, Fruchart J-C, Besnard S (2001). Transcriptional regulation of *Apolipoprotein C*-*III* gene expression by the orphan nuclear receptor RORalpha. J Biol Chem.

[41] Lau P, Bailey P, Dowhan DH, Muscat GEO (1999). Exogenous expression of a dominant negative RORα1 vector in muscle cells impairs differentiation: rORα1 directly interacts with p300 and MyoD. Nucleic Acids Res.

[42] Guo B, Greenwood PL, Cafe ML, Zhou G, Zhang W, Dalrymple BP (2015). Transcriptome analysis of cattle muscle identifies potential markers for skeletal muscle growth rate and major cell types. BMC Genomics.

[43] Youn HD, Grozinger CM, Liu JO (2000). Calcium regulates transcriptional repression of myocyte enhancer factor 2 by histone deacetylase 4. J Biol Chem.

[44] McKinsey TA, Zhang CL, Olson EN (2000). Activation of the myocyte enhancer factor-2 transcription factor by calcium/calmodulin-dependent protein kinase-stimulated binding of 14-3-3 to histone deacetylase 5. Proc Natl Acad Sci USA.

[45] Gu Z, Eleswarapu S, Jiang H (2007). Identification and characterization of microRNAs from the bovine adipose tissue and mammary gland. FEBS Lett.

[46] Hudson NJ, Reverter A, Wang Y, Greenwood PL, Dalrymple BP (2009). Inferring the transcriptional landscape of bovine skeletal muscle by integrating co-expression networks. PLoS One.

[47] Alfieri CM, Evans-Anderson HJ, Yutzey KE (2007). Developmental regulation of the mouse *IGF*-*I* exon 1 promoter region by calcineurin activation of *NFAT* in skeletal muscle. Am J Physiol Cell Physiol.

[48] Yang TT, Suk HY, Yang X, Olabisi O, Yu RY, Durand J (2006). Role of transcription factor NFAT in glucose and insulin homeostasis. Mol Cell Biol.

[49] Bae SC, Lee KS, Zhang YW, Ito Y (2001). Intimate relationship between TGF-Î^2^/BMP signaling and runt domain transcription factor, PEBP2/CBF. J Bone Joint Surg Am..

[50] Li S, Czubryt MP, McAnally J, Bassel-Duby R, Richardson JA, Wiebel FF (2005). Requirement for serum response factor for skeletal muscle growth and maturation revealed by tissue-specific gene deletion in mice. Proc Natl Acad Sci USA.

[51] Hsieh CL, Liu H, Huang Y, Kang L, Chen HW, Chen YT (2014). ADAR1 deaminase contributes to scheduled skeletal myogenesis progression via stage-specific functions. Cell Death Differ.

[52] Jiao Y, Bishop CE, Lu B (2012). *Mex3c* regulates insulin-like growth factor 1 (IGF1) expression and promotes postnatal growth. Mol Biol Cell.

[53] Jiao Y, George SK, Zhao Q, Hulver MW, Hutson SM, Bishop CE (2012). *Mex3c* mutation reduces adiposity and increases energy expenditure. Mol Cell Biol.

[54] Gu Q, Nagaraj SH, Hudson NJ, Dalrymple BP, Reverter A (2011). Genome-wide patterns of promoter sharing and co-expression in bovine skeletal muscle. BMC Genomics.

[55] Maier R, Moser G, Chen GB, Ripke S, Coryell W, Cross-Disorder Working Group of the Psychiatric Genomics Consortium (2015). Joint analysis of psychiatric disorders increases accuracy of risk prediction for schizophrenia, bipolar disorder, and major depressive disorder. Am J Hum Genet.

[56] Maher B (2012). ENCODE: the human encyclopaedia. Nature.

[57] Renand G, Fisher AV (1997). Comparison of methods for estimating carcass fat content of young Charolais bulls in performance testing station. Livest Prod Sci.

