# Supplementary figures and images for: Multi-breed and multi-trait co-association analysis of meat tenderness and other meat quality traits in three French beef cattle breeds
Source: Genet Sel Evol. 2016 Apr 23;48:37. doi: 10.1186/s12711-016-0216-y (PMC4842279; doi:10.1186/s12711-016-0216-y)

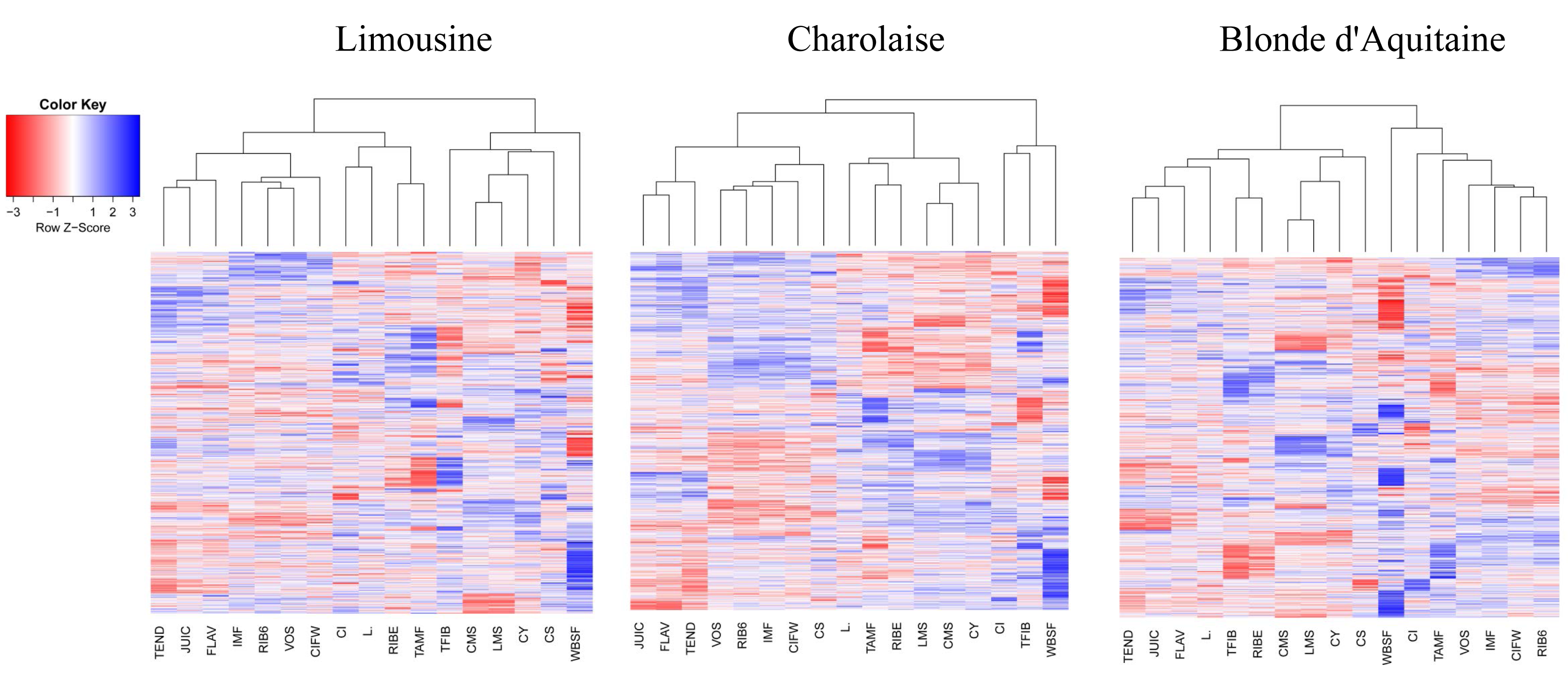

Supplement: Supplementary file 1 — 10.1186/s12711-016-0216-y Hierarchical cluster analysis for the Limousine, Charolaise and Blonde d'Aquitaine breeds. The figure represents the distribution of clusters for the 17 traits analyzed on the x-axis and the distribution of clusters for co-associated genes on the y-axis. Abbreviations for traits are described in Table 1. [file 12711_2016_216_MOESM1_ESM.tif]

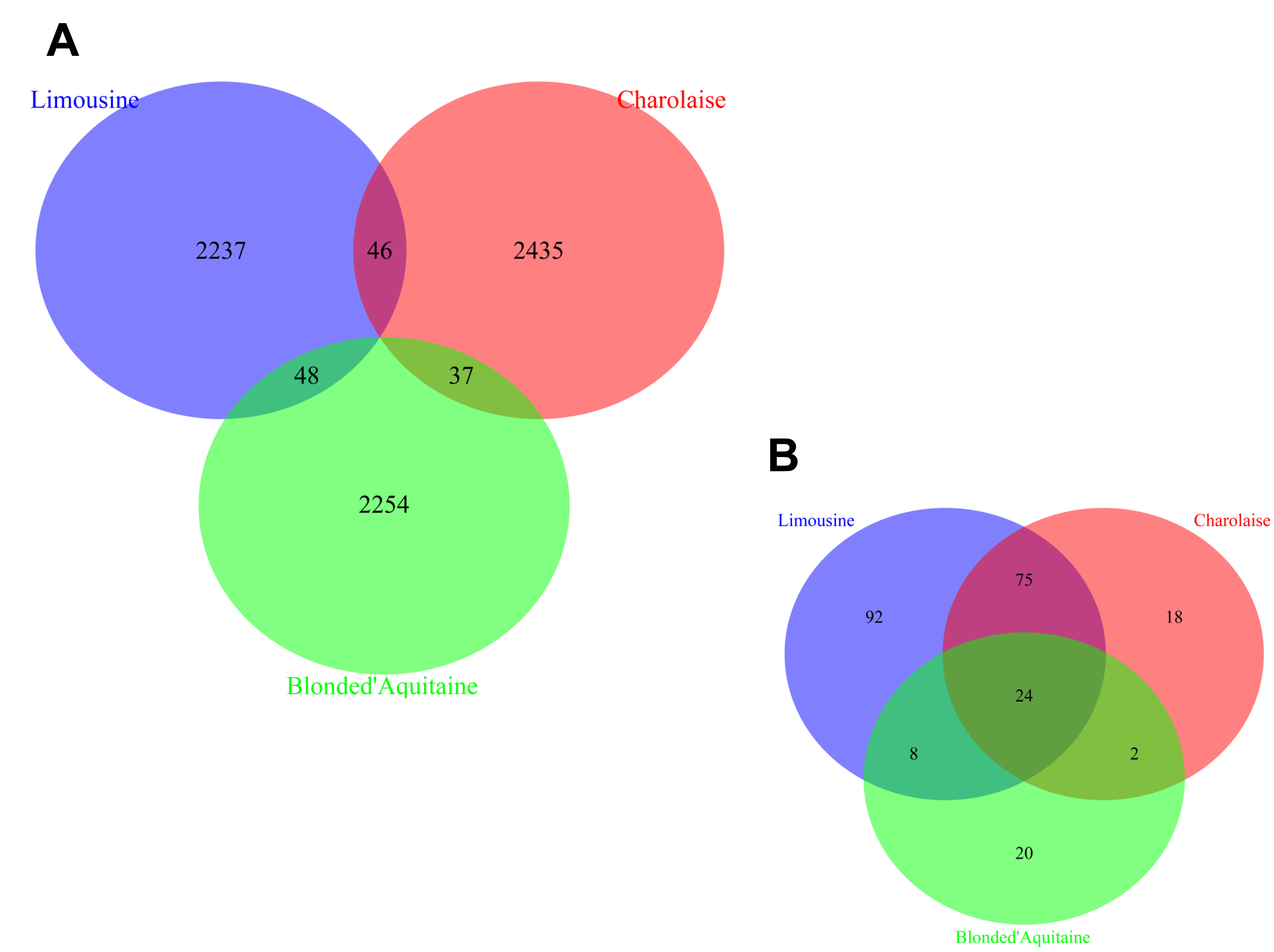

Supplement: Supplementary file 3 — 10.1186/s12711-016-0216-y (A) Number of overlapping SNPs. (B) Overlapping pathways. The figure represents the number of overlapping SNPs and pathways identified across the three beef cattle breeds. [file 12711_2016_216_MOESM3_ESM.tif]
